# Prior Mental Fatigue Impairs Marksmanship Decision Performance

**DOI:** 10.3389/fphys.2017.00680

**Published:** 2017-09-08

**Authors:** James Head, Matthew S. Tenan, Andrew J. Tweedell, Michael E. LaFiandra, Frank Morelli, Kyle M. Wilson, Samson V. Ortega, William S. Helton

**Affiliations:** ^1^United States Army Research Laboratory, Human Research and Engineering Directorate, Dismounted Soldier and Team Performance Branch Aberdeen Proving Ground, MD, United States; ^2^United States Army Research Laboratory, Human Research and Engineering Directorate, Integrated Capability Enhancement Branch Aberdeen Proving Ground, MD, United States; ^3^Psychology Department, University of Huddersfield Huddersfield, United Kingdom; ^4^ARCH Lab, Human Factors and Applied Cognition, George Mason University Fairfax, VA, United States

**Keywords:** fine-motor performance, marksmanship decision accuracy, live-fire, response inhibition intervention, HRV, soldiers

## Abstract

**Purpose:** Mental fatigue has been shown to impair subsequent physical performance in continuous and discontinuous exercise. However, its influence on subsequent fine-motor performance in an applied setting (e.g., marksmanship for trained soldiers) is relatively unknown. The purpose of this study was to investigate whether prior mental fatigue influences subsequent marksmanship performance as measured by shooting accuracy and judgment of soldiers in a live-fire scenario.

**Methods:** Twenty trained infantry soldiers engaged targets after completing either a mental fatigue or control intervention in a repeated measure design. Heart rate variability and the NASA-TLX were used to gauge physiological and subjective effects of the interventions. Target hit proportion, projectile group accuracy, and precision were used to measure marksmanship accuracy. Marksmanship accuracy was assessed by measuring bullet group accuracy (i.e., how close a group of shots are relative to center of mass) and bullet group precision (i.e., how close are each individual shot to each other). Additionally, marksmanship decision accuracy (correctly shooting vs. correctly withholding shot) when engaging targets was used to examine marksmanship performance.

**Results:** Soldiers rated the mentally fatiguing task (59.88 ± 23.7) as having greater mental workload relative to the control intervention [31.29 ± 12.3, *t*_(19)_ = 1.72, *p* < 0.001]. Additionally, soldiers completing the mental fatigue intervention (96.04 ± = 37.1) also had lower time-domain (standard deviation of normal to normal R-R intervals) heart rate variability relative to the control [134.39 ± 47.4, *t*_(18)_ = 3.59, *p* < 0.001]. Projectile group accuracy and group precision failed to show differences between interventions [*t*_(19)_ = 0.98, *p* = 0.34, *t*_(19)_ = 0.18, *p* = 0.87, respectively]. Marksmanship decision errors significantly increased after soldiers completed the mental fatigue intervention (48% ± 22.4) relative to the control intervention [*M* = 32% ± 79.9, *t*_(19)_ = 4.39, *p* < 0.001]. There was a significant negative correlation between shooting response time and errors of commission (*r* = −0.61; *p* = 0.004) when preceded by the mental fatigue intervention, but not the control (*r* = −0.31; *p* = 0.17).

**Conclusion:** The mental fatigue intervention was successful in eliciting fatigue which was supported subjectively and objectively. Marksmanship judgment performance is significantly reduced when soldiers are mentally fatigued, although shot accuracy is not.

## Introduction

Mental fatigue is the psychophysiological response experienced by an individual who is exposed to a mentally demanding task which results in the subjective feeling of “tiredness” and “lack of energy” (Boksem and Tops, 2008; Marcora et al., 2009). For example, individuals engaging in executive function tasks such as vigilance (e.g., sustained attention task) or response inhibition task (e.g., Sustained Attention to Response Task and Stoop Task; SART) often self-report mental fatigue which can subsequently influence physical performance (Pageaux et al., 2014). Mental fatigue has been shown to impair performance in civilian occupations such as commercial airline pilots, baggage inspection, power plant operators, and medical occupations (Warm et al., 2008).

Mental fatigue has been shown to have measurable influence on subsequent gross-motor performance. Completing a mental fatiguing task (e.g., response inhibition and vigilance task) prior to physical exercise affects physical performance. For example, participants completing a cycling task reach exhaustion sooner or complete less distance in a given time when mentally fatigued prior to the cycling task than when not mentally fatigued (Marcora et al., 2009; Pageaux et al., 2014). Indeed, a recent investigation in our laboratory has further corroborated that this phenomena is not task specific to isolated stationary tasks such cycle ergometers or treadmills, but also tasks involving full-body resistance exercise (Head et al., 2016).

The influence of prior mental fatigue on physical performance may also influence performance on tasks that are relatively more fine-motor by nature. For example, Rozand et al. (2016) have shown that prior mental fatigue influences performance on an Arm-pointing task. The Arm-pointing task requires participants to use a stylus instrument (i.e., similar to a pen) with their dominate hand and make contact within two separated predesignated squares. The Arm-pointing task requires participants to internally balance between speed and accuracy. Rozand et al. found measurable performance impairments in the form of slower response speed as a function of mental fatigue. Although this finding may lack applicability to real-world tasks, it does suggest mental fatigue may influence relatively more applied fine-motor tasks such as marksmanship.

Small arms marksmanship is a fine-motor task that is relevant to both sport and military occupations. Marksmanship is a complex task in which performance can be modified by both physiological and psychological factors (Helin et al., 1987; Konttinen et al., 1998; McDermott et al., 2001; Lakie, 2010). Indeed, sports like Olympic biathlons require athletes to precisely engage targets while physically fatigued; however, participants may also experience cognitive influences such as anxiety and stress (Vickers and Williams, 2007). Marksmanship performance in military and law enforcement settings can also be influenced by cognitive factors, but in these cases have the additional factor of grave consequences (e.g., deadly force judgment) (Johnson et al., 2014).

Marksmanship accuracy is influenced by a myriad of factors including but not limited to environment (hot vs. cold) (Lakie and Campbell, 1995), stress (Solberg et al., 1996), and physical fatigue (Tenan et al., 2017). Prior research conducted at U. S. Army Research Institute of Environmental Medicine has provided evidence that marksmanship performance is influenced by mental fatigue (Johnson and Merullo, 1999). In Johnson and Merullo's investigation, participants completed a mentally fatiguing simulated sentry marksmanship task for 3 h whereby they made friend-foe discriminations. Through a double-blind design, participants received either a placebo or stimulant (caffeine). As predicted, participants receiving the stimulant had significantly decreased friend-foe decision errors as a function of time-on-task. This improved performance in the sentry task was attributed to the caffeine mitigating the mental fatigue experienced by the participants.

Interestingly, recent investigations have shown a potential relationship between mental fatigue and friend-foe decision accuracy as indexed by fine-motor control (Wilson et al., 2015). Specifically, the authors found that participants shooting in a target rich environment (high-shoot/low no-shoot) had significant increases in errors of commission (i.e., incorrectly shooting instead of withholding shot) and speeded responses resulting in speed accuracy trade-offs (SATO). The authors argue that these errors of commission are a result of participants developing a feed-forward motor ballistic routine which becomes difficult to readily inhibit. Thus, participants are often fully aware of seeing the no-shoot stimuli but accidently respond due to the motor ballistic routine (Head and Helton, 2012, 2013). This failure in fine-motor control has been attributed to the supervisory attentional system being overloaded (Helton, 2009). In other words, mental fatigue appears to modulate fine-motor control and influence how participants respond to targets.

The likelihood of soldiers developing mental fatigue in the field has only increased with the advent of head-up displays and changing battlefield scenarios [e.g., constant scanning for improvised explosive devices (IEDs)]. For example, prior investigations have shown that monitoring stimuli over time can increase mental fatigue which consequently decreases sensitivity to identify targets (e.g., threats) in an environment (Mackworth, 1948). Moreover, previous investigations have established that lapses in target identification in a military and civilian context can have dire consequences (Wilson et al., 2015). Given the unpredictability of war, a soldier may be required to engage in a firefight at a moment's notice after monitoring and processing information for long periods of time resulting in mental fatigue.

In the current investigation, we seek to better understand whether mental fatigue influences marksmanship performance in a live-fire scenario. As noted, there is a lack of ecological validity and applicability of laboratory findings with concern to mental fatigue on subsequent physical performance see Duncan et al. (2015). Indeed, only recently have investigations begun to address the influence of mental fatigue on real world task performance (e.g., soccer performance, cycling, and running) (Marcora et al., 2009; Pageaux et al., 2014; Smith et al., 2016). Thus, in the current investigation we utilize an open air acoustic location-of-miss-and-hit live fire range to evaluate how mental fatigue impairs marksmanship performance in an experimentally controlled live-fire scenario. It was hypothesized that inducing mental fatigue in soldiers will impair marksmanship performance (shooting accuracy and marksmanship decision accuracy).

## Methods

### Participants

Twenty healthy male soldiers participated in all phases of experimentation. Soldiers provided written informed consent in accordance with the Helsinki Accord and ethics permission was obtained from the U.S. Army Research Laboratory Institutional Research Board. All soldiers were either 11B (infantry) or 11C (indirect fire infantry) which have extensive training in rifle marksmanship. Each soldier was required to have qualified with a rifle in the Army basic marksmanship training course within the past year. The Army basic marksmanship course involves soldiers engaging pop-up targets at varying distances (5–300 m). Marksman qualifications are a function of number of targets hit out of 40 shots (e.g., Marksman 23–29, Sharpshooter 30–35, and Expert 36–40). Given the emphasis on marksmanship for infantry, the qualification scores were relatively skewed with 68% of soldiers reporting Expert qualification, 28% Sharpshooter, and 4% Marksman.

### Procedure

Soldiers were recruited from various duty stations within the continental United States. All soldiers completed a control (passive video watching) and a mentally fatiguing (response inhibition task) intervention prior to performing a marksmanship task, in a randomized and counterbalanced design. Soldiers completed three visits which took place in a single week (once a day) at the same time of day for each participant in an isolated room and shooting lane at the Aberdeen Proving Ground shooting range. Soldiers were given specific instructions to sleep for at least 7 h and were also instructed to be consistent with their stimulant and depressant intake each day (i.e., caffeine, nicotine, and alcohol). Substance use was recorded each day prior to starting the experimental tasks. *Post-hoc* analysis did not reveal any relationship between performance metrics/heart rate variability and substance use.

**Figure 1:**
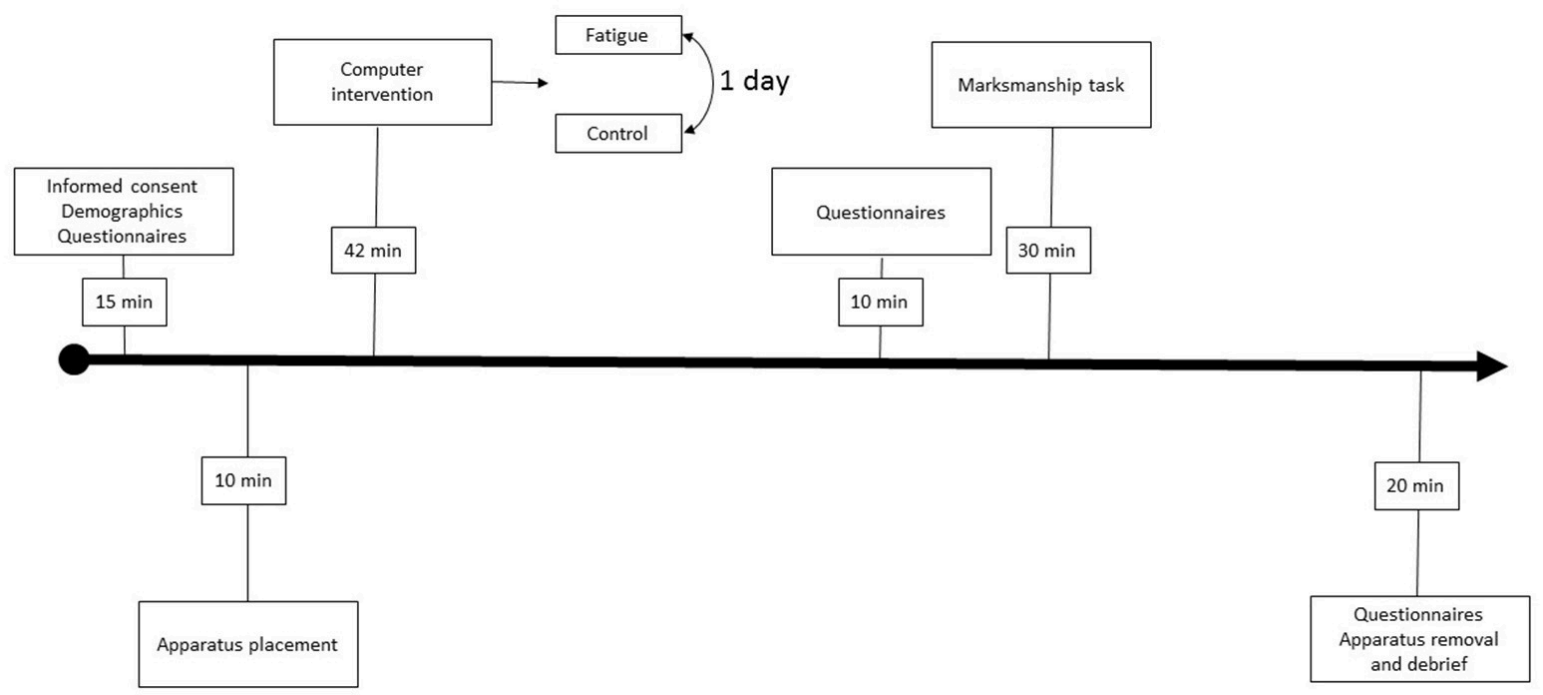
Overview of study excluding practice day.

Upon arrival on day one, soldiers first completed an informed consent, demographics survey, and practiced the computer and shooting task to be completed on subsequent days. For day two and three, soldiers were equipped with a wireless five-lead electrocardiogram (ECG; Shimmer, Dublin, Ireland). To enhance external validity and safety, soldiers were required to wear an Improved Outer Tactile Vest, Advanced Combat Helmet and seeing/hearing protection. Soldiers were only required to wear the seeing/hearing protection during the shooting portion of the study. Once outfitted with the protective equipment and ECG unit, soldiers completed the experimental or control intervention (i.e., mental fatigue or video watching, respectively). After the experimental or control intervention, participants completed the National Aeronautical and Space Administration Task Load Index [NASA-TLX; Hart and Staveland (1988)]. Lastly, soldiers rated the perceived workload of each shooting scenario repeatedly with the NASA-TLX. See Figure 1 for outline of study.

### Mental fatigue intervention

Prior investigations on this topic have utilized response inhibition tasks to elicit mental fatigue prior to a physical task (Pageaux et al., 2014). Thus, in the current investigation soldiers completed the Sustained Attention to Response Task. The SART has been used frequently to measure response inhibition and is fatiguing (Head and Helton, 2012). The mental fatigue intervention took place in an isolated room. Soldiers were seated 50 cm in front of a video display terminal (53.4 × 33 cm, 60 Hz refresh rate) which was mounted at eye level. Soldiers' head movements were not restrained. Time-keeping devices such as watches and cell phones were surrendered at the start of the task. Soldiers completed a SART (high go/ low No-go) monitoring for numeric stimuli (1–9). Soldiers were instructed to respond as fast and accurately as possible to the target number 1–9, except for 3. Prior to the start of the task, soldiers completed a practice trial where they received feedback on their performance. Soldiers completed this practice on day one and also again before they completed the task on day 2 and 3. The SART was 49 min in duration and was comprised of 4 periods of watch, 12.25 min in length. Numeric digits were all the same font (Courier); however, font size varied between 48, 72, 94, 100, and 120, with height varying between 12 and 29 mm. Each trial consisted of a single digit presented centrally on the screen for 250 ms followed immediately by a 900 ms mask. Soldiers were instructed to respond with their index finger on their dominant hand using a response box. Errors of commission (inappropriately responding to No-go stimuli), errors of omission (inappropriately withholding response to go stimuli), and response time to correct go stimuli were calculated for each Soldier per period of watch.

### Control intervention

The computer screen from the SART was used to present a 49 min video train documentary. The documentary was “The American Orient Express” (Pegasus-Eagle Rock Entertainment, 2004) which consisted of footage about trains and travel. This type of stimuli has been used in similar studies due to the neutral content maintaining stable mood and heart rate (Dennis et al., 2015; Head et al., 2016).

### Marksmanship task

Soldiers were armed with a Heckler & Koch automatic 416 subcompact carbine (HK-416 A5). Target sighting was achieved using a holographic optic with a collimated red dot reticle (EOTech EXPS3, 1X power). The shooting task took place at the U.S. Army Research Laboratory Shooter Performance Research Facility (M-Range) at Aberdeen Proving Ground, Maryland. Soldiers were stationed in a fire trench (i.e., foxhole) with a range safety personnel.

**Figure 2:**
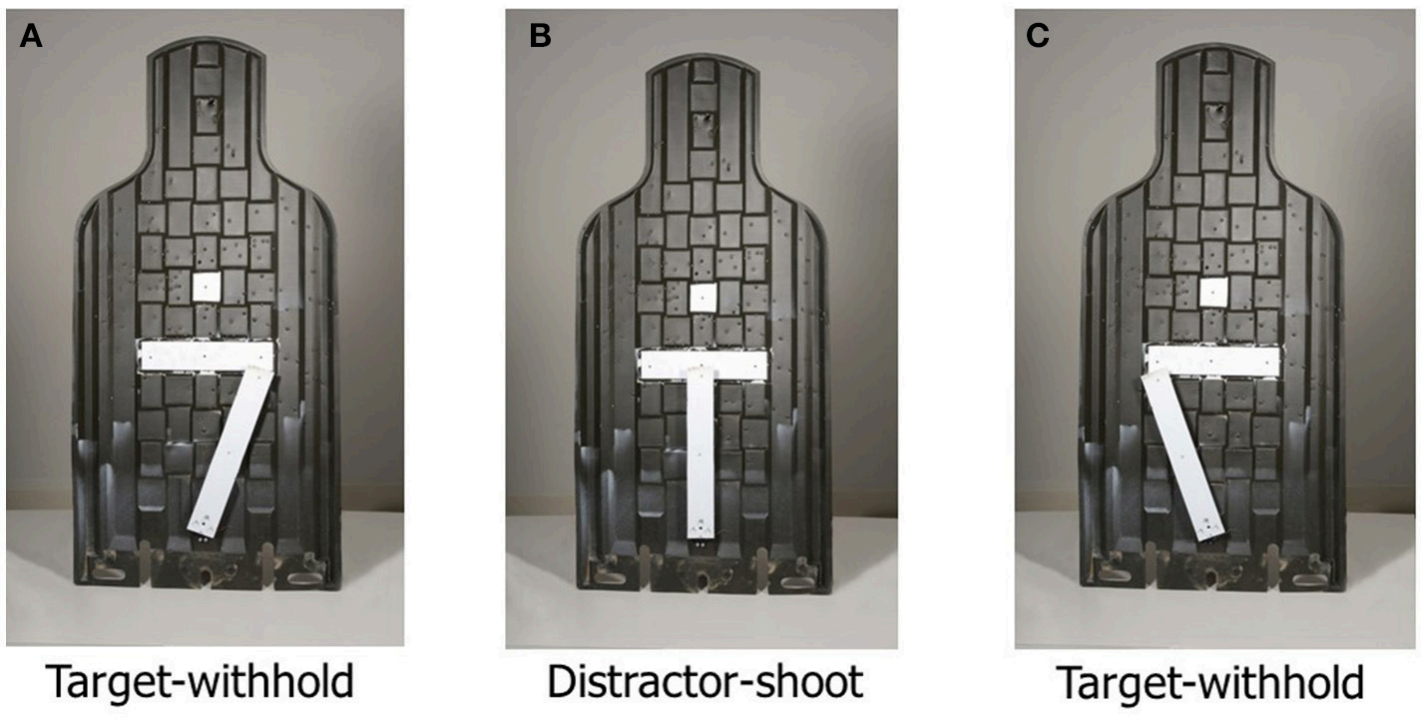
E-silhouette targets [45° **(A)**, 0° **(B)**, and 135° **(C)**] positions left to right.

During the marksmanship task, soldiers were instructed to be in a high-ready shooting position with their rifle resting on sandbags in the foxhole. Soldiers were instructed that they would be monitoring a single shooting lane and shooting predefined E-silhouette targets. An E-silhouette is a dark green target (49.5 × 101.6 cm) that is commonly used by the U.S. military for marksmanship training (see Figure 2). The shooting lane contained three E-silhouette targets positioned 25, 50, and 100 m behind a grass berm in front of the participant. E-silhouettes were affixed on mechanical arm that permitted the E-silhouette targets to rapidly be raised or lowered above the grass berm according to a predefined shooting scenario. Each E-silhouette target was outfitted with a horizontal (6 × 26 cm) and vertical (5 × 32 cm) white line. The horizontal white line was fixed; however, the vertical white line was a moving appendage that was affixed to a servo motor which permitted it to move between 135, 0, and 45° (see Figures 2A–C). Prior to the shooting scenario beginning, soldiers were instructed to load their rifle with a 30 round magazine and chamber a round. Soldiers were instructed to shoot E-silhouette targets with a single shot when the appendage was in the 90° position whereby the white lines made a “T” shape (see Figure 2B). Conversely, soldiers were instructed to withhold their response when the vertical horizontal white line was either in the 135° or 45° position (see Figures 2A,B). To control for target presentation, participants completed shooting scenarios whereby the target location was predictable vs. unpredictable. Order of shooting scenarios was counterbalanced across soldiers. In the predictable scenario, E-silhouette targets would raise up in a predictable sequence (25, 50, and 100 m) repeatedly; however, whether the E-silhouette was a target to shoot or not shoot was random. In the unpredictable shooting scenario, E-silhouette targets would randomly occur in one of the target distances; however, probability of occurrence was equal for each distance.

The marksmanship task is based on the SART and has been used in previous simulated marksmanship task (Wilson et al., 2013). The marksmanship task is a high-shoot, low no-shoot target detection task which requires active response inhibition. Soldiers were exposed to eight shoot targets and two no-shoot targets per each target distance which resulted in a no-shoot target probability of 20% and a shoot probability of 80%. In total, soldiers completed four trials of each shooting condition. Soldiers were instructed by the safety personnel to reload their weapon and chamber a new round between each trial performance on all shooting conditions (predictable vs. unpredictable). Target silhouettes exposure was 600 ms followed by a 2,500 ms inter-stimulus interval. Thus, each trial was ~1.6 min in duration. Shots taken during no-shoot target exposure and inter-stimulus interval were recorded as errors of commission. Soldiers were instructed to shoot as fast and accurately as possible. Conversely, shoot targets not responded to within the same timeframe were recorded as errors of omission. Soldiers only received feedback on marksmanship decision accuracy (shoot vs. no-shoot errors) during the practice session.

Marksmanship performance for military personnel is multifaceted which includes target accuracy and marksmanship decision accuracy (i.e., shoot vs. no-shoot) (Johnson et al., 2014; Wilson et al., 2015). To assess target accuracy; target hit proportion, distance of the center of the shot group (DCSG) relative to target center, correct shot response time, and shot group precision (SGP) was calculated for each soldier (Grubbs, 1964). DCSG is used to index how close a group of shots are relative to the center aiming point of a target (i.e., shot group accuracy). SGP indexes how close (i.e., proximity of each individual shot) a group of shots are to each other. In the current investigation, a high-go/low no-go shooting paradigm was utilized (Wilson et al., 2015). This paradigm permits the measurement of marksmanship accuracy and marksmanship decision accuracy. Failures in marksmanship decision accuracy have been attributed to fine-motor response failures that is typified by a negative correlation between response time and errors of commission (SATO) (Wilson et al., 2015).

### Psychological scales

The NASA-TLX is a workload measure composed of 6-items (Hart and Staveland, 1988). The questionnaire contains three items that measure external demand (mental, temporal, and physical) and an additional three items (effort, performance, and frustration) that measure internal responses to the external demands. The six items were aggregated together for a composite global workload score. The NASA-TLX was given after each intervention and shooting scenario in the marksmanship task.

### Electrocardiogram instrumentation and analysis

A 5-lead ECG (Shimmer3, Shimmer, Ireland, UK) was instrumented on the soldier to record ECG waveforms during mental tasks. Electrode placement was confirmed by visually inspecting the ECG waveform in real time on a wireless tablet (Samsung Galaxy 10, Suwon, SK) prior to continuation. The ECG signal was sampled at 1,024 Hz and recorded for off-line analysis. The R-R intervals of the ECG waveform were later determined algorithmically (Goldberger et al., 2000) and visually confirmed by at least one researcher to ensure accurate interval identification. Specifically, only the last 5 min of the mental and control intervention were recorded and analyzed using the RHRV package, version 4.2.2 (Rodríguez-Liñares et al., 2008) to calculate the standard deviation of the R-R intervals standard deviation NN interval (SDNN). The SDNN calculation was used to gauge HRV as a function of the intervention type. The HRV was used as a manipulation check to verify whether participants had an autonomic stress response to the mentally fatiguing task (Luque-Casado et al., 2016).

### Statistical analysis

Mean and standard deviation of the mean are reported unless otherwise stated. Pearson r correlations were conducted for SATO measures (errors of commission and response time) for the marksmanship task. All omnibus analyses were performed via mixed-effects models (unstructured variance/covariance structure) to account for the repeated measure design. Soldiers were treated as random effects using restricted maximum likelihood for each analysis. Residuals were inspected for normality and the assumption of constant variance was verified by plotting the residuals against the fitted data. Cook's d influence diagnostics revealed one subject as a potential outlier. However, removal of the subject did not substantially alter model estimates and did not alter variable significance. Therefore, the soldier was retained for further analysis. All statistical analysis were performed in R-3.2.3 (R Development Core Team, 2015) using lmerTest (Trottmann et al., 2016).

## Results

### Interventions

Soldiers had significantly longer correct responses to stimuli as a function of time during the mental fatigue intervention [*F*_(3, 63)_ = 3.88, *p* = 0.01], see Table 1. Additionally, soldiers had significantly more errors of omission (incorrectly withholding to distractor) as a function of time [*F*_(3, 63)_ = 11.57, *p* < 0.001]. However, errors of commission (inappropriately responding to go target) did not change as a function of time [*F*_(3, 63)_ = 1.52, *p* = 0.21, see Table 1]. Soldiers completing the mental fatigue intervention (96.04 ± 37.1) had significantly lower HRV (SDNN) relative to the control intervention [134.39 ± 47.4, *t*_(18)_ = 3.59, *p* < 0.001]. For the subjective workload measure, soldiers rated the fatigue intervention (59.88 ± 15.2) as having greater mental workload relative to the control intervention [31.29 ± 15.6, *t*_(19)_ = 7.71, *p* < 0.001].

**Table 1:** Descriptive statistics (M; SD) for the mental fatigue intervention.

**Time period**	**1**	**2**	**3**	**4**
Errors of commission (%)	3.00 (9.06)	4.08 (9.00)	5.05 (9.00)	6.00 (9.00)
Errors of omission (%)	10.00 (13.02)	13.00 (9.02)	16.00 (9.02)	19.00 (9.02)
Response time (msec)	886 (93.06)	847 (60.45)	849 (60.43)	872 (60.43)

*Performance on the mental fatigue intervention as a function of time*.

### Marksmanship performance

**Figure 3:**
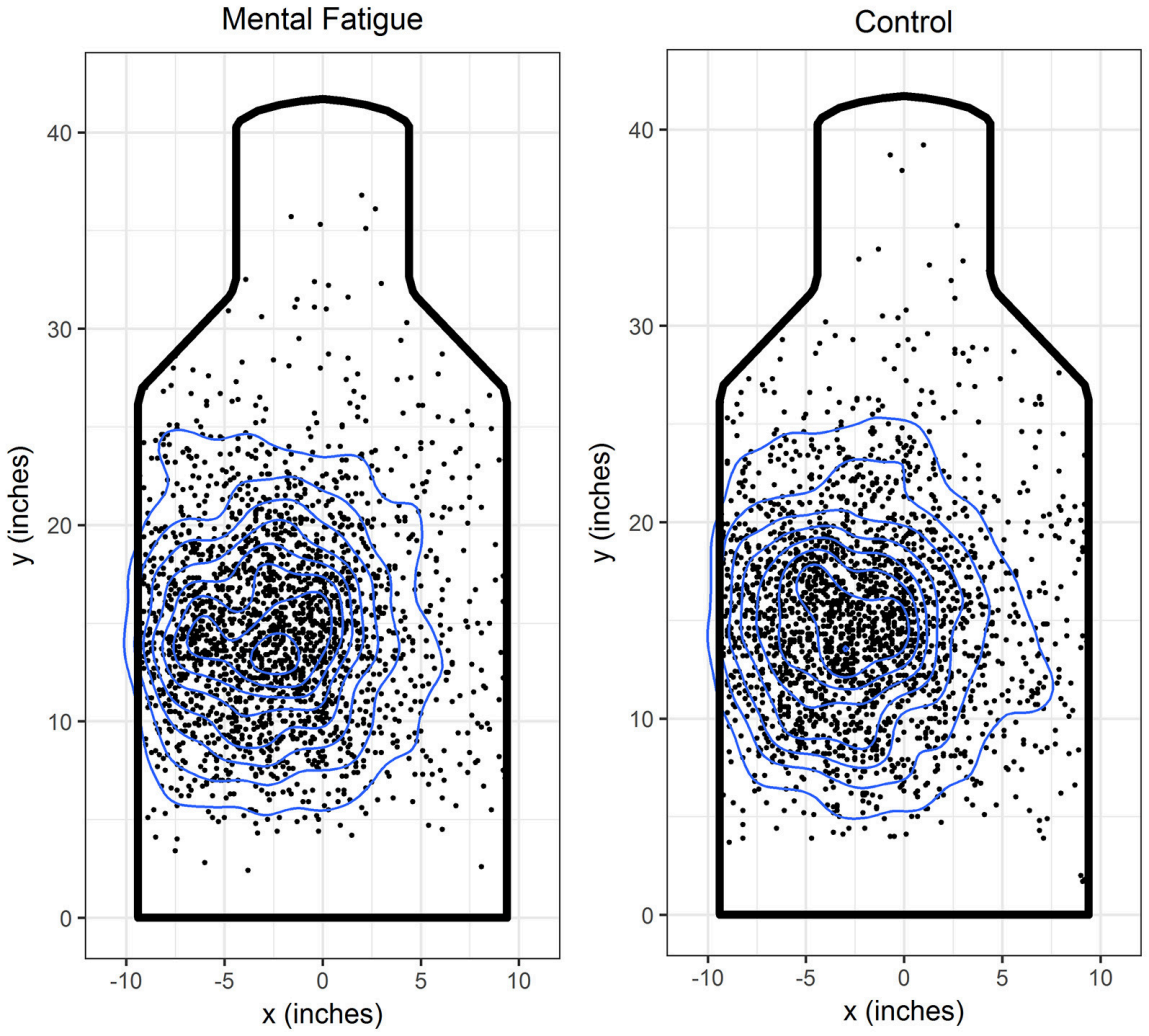
Individual coordinates for each projectile which hit the e-silhouette target during the marksmanship task preceded by mental fatigue and control intervention are plotted with a two-dimensional kernel density plot where higher densities are contained within lower densities.

All omnibus significance tests and descriptive statistics related to the marksmanship task are displayed in Table 2. Soldiers did not show a significant difference for response time as a function of intervention. Additionally, soldiers did not show a significant difference in marksmanship accuracy for hit proportion, DCSG, and SGP. Visual inspection of two-dimensional kernel density plots further supports that the intervention did not influence shot accuracy or precision (see Figure 3). Soldiers exhibited a significant negative correlation between response time and errors of commission after the mental fatigue (*r* = −0.62, *p* = 0.004) intervention, but not the control (*r* = −0.31, *p* = 0.17). Soldiers completing the mental fatigue intervention exhibited a significant increase in marksmanship decision accuracy errors (i.e., increased errors of commission) relative to the control intervention (see Table 2). Response time and errors of omission failed to reach significance. For the subjective workload measure (NASA-TLX), soldiers did not rate marksmanship task with a greater workload rating as a function of intervention type (fatigue vs. control).

**Table 2:** Descriptive statistics (M; SD) and significance test for marksmanship task and workload measure.

	**Fatigue**	**Control**	**Significance test**
Hit proportion (%)	76.44 (8.98)	78.45 (8.50)	*t*_(19)_ = 0.58, *p =*.57
DCSG (cm)	37.72 (3.81)	38.56 (4.63)	*t*_(19)_ = 0.98, *p =* 0.34
SGP (cm)	13.18 (1.32)	13.13 (1.31)	*t*_(19)_ = 0.18, *p =* 0.86
Errors of commission (%)	48.05 (22.42)	32 (17.94)	*t*_(19)_ = 4.39, *p =* 0.001
Errors of omission (%)	7.25 (7.65)	6.26 (6.74)	*t*_(19)_ = 0.67, *p =* 0.51
Response time (ms)	791.87 (65.52)	794.50 (47.48)	*t*_(19)_ = 0.*25, p =* 0.81
Workload score	89.35 (23.71)	94.04 (12.35)	*t*_(19)_ = 1.72, *p =* 0.10

*Performance and workload score on the marksmanship task as a function of intervention type. DCSG, Distance of the center of the shot group; SGP, Shot group precision*.

## Discussion

Prior investigations have provided evidence that mental fatigue impairs both gross-motor (Head et al., 2016) and fine-motor physical performance (Duncan et al., 2015) within a laboratory setting. However, the current investigation provided mixed results concerning the influence of mental fatigue on subsequent marksmanship performance in a live-fire scenario. The present study did not show a significant difference in marksmanship accuracy (hit proportion, DCSG, and SGP) or correct hit response time when preceded by the mental fatigue or control intervention. However, there was a significant effect of mental fatigue on marksmanship decision accuracy as indexed cognitively as fine-motor control.

### Mental fatigue task

Prior investigations on the topic of mental fatigue and its influence on subsequent physical performance have utilized various methods to elicit mental fatigue. These methods have included tasks that involve selective and sustained attention (e.g., Continuous Performance Task; CPT) and response inhibition (i.e., Stroop task). The measurable physical performance impairments caused by the CPT and Stroop task have been attributed to these tasks influencing the anterior cingulate cortex area of the brain which has been associated with processing perceived effort (Critchley et al., 2000; Williamson et al., 2003). Generally, these performance impairments have been noted to range between 2.3 and 17.8% (see Head et al., 2016). In the current investigation, soldiers' error rate in the form of errors of commission (incorrectly shooting instead of withholding) was significantly greater in magnitude relative to prior investigations. Specifically, soldiers had a 33% increase in error of commission rate when cognitive fatigued relative to the control. Relative to prior investigations, this pronounced increased error of magnitude may be attributed to the fact that both the mentally fatiguing task and the marksmanship task both required active response inhibition. In other words, unlike prior investigations, the mentally fatiguing task and the subsequent physical task arguably shared the same cognitive resources (McCracken and Aldrich, 1984; Wickens, 2002). Thus, after soldiers completed the mentally fatiguing computer task, they completed a physical task that required the same cognitive process (response inhibition) which likely compounded the effects of fatigue on performance.

Converging evidence is provided behaviorally, subjectively, and physiologically that the mental fatigue task was fatiguing in the current investigation. Generally, performance on the SART is characterized by speeded responses and increased errors of commission (Head and Helton, 2012, 2013). However, in the current investigation soldiers exhibited slower response times and increased errors of omission to go targets as a function of time-on-task during the SART. This result is likely due to the nature and duration length of the SART selected for the current investigation.

Due to the highly repetitive nature of the SART, participants responding to frequent go targets often develop a feed-forward motor ballistic routine which becomes difficult for the central executive system to inhibit. This difficulty in inhibition has been attributed to overloading the central executive system (i.e., mental fatigue) (Helton et al., 2010). A proposed adaptive response (i.e., replenish mental resources) to alleviate mental fatigue during the SART is strategic forced rest breaks (errors of omission) which is analogous to taking a mental breather (Stevenson et al., 2011). Participants exhibited a greater amount of errors of omission during the last period of the computer task relative to the start. Given the fatiguing nature of the task and the duration (49 min) participants were likely taking increased forced rest breaks in an attempt to mitigate the experienced mental fatigue.

Both objective and subjective measures provided support that the response inhibition task was successful in eliciting mental fatigue. First, participants had lower HRV during the last 5 min of the mental fatigue intervention relative to the control intervention. As discussed prior, lower HRV is associated with a decreased parasympathetic response to stress (Kakutani et al., 1991). Second, after completing the interventions, participants subjectively rated the mental fatigue intervention as having a greater global workload relative to the control intervention. Collectively, the mental fatiguing task was stressful and perceived as being fatiguing by the soldiers.

### Effects of mental fatigue on marksmanship performance

Mental fatigue did not significantly influence any of marksmanship accuracy metrics commonly used to index the marksmanship performance (Grubbs, 1964). Although soldiers were shooting from a standing supported position, visual inspection of individual shots coupled with the accuracy and precision measures indicate that soldiers' shots were widely dispersed. This is likely due to the temporally demanding nature of the shooting paradigm (Wilson et al., 2015). Soldiers had to quickly scan their environment, acquire the target in their sights, and execute a trigger pull. Given the temporal demand of the shooting task, soldiers likely had difficulty hitting the center of the target. Future investigations should consider examining marksmanship tasks that require fine-motor control but are slower in nature (e.g., long range precision target engagement). The decreased temporal demand may alleviate the difficulty of participants acquiring the target which would enable a more clear understanding of the influence of prior mental fatigue on marksmanship accuracy.

The current investigation utilized a high-go/low-no-go shooting task previously used in a simulated marksmanship task (Wilson et al., 2015). Overall, there was a relatively high error of commission rate (40%) regardless of prior mental fatigue or not. This pronounced error rate on this task has been attributed to failures of inhibitory control due to the highly repetitive nature of the task (i.e., numerous go responses) (Helton, 2009). Conversely, the overall error of omission rate was relatively low (7%). Given the short duration of each trial (1.6 min) it is likely that soldiers did not require forced rest breaks as found in high-go/low no-go tasks of longer durations (Stevenson et al., 2011).

As predicted, marksmanship decision accuracy was significantly impaired (increased errors of commission) by the mental fatigue intervention in a live-fire scenario as found in simulations (Johnson and Merullo, 1999; Wilson et al., 2015). Interestingly, unlike Rozand and colleagues findings, soldiers exhibited speeded responses in the marksmanship task as function of the mental fatigue intervention instead of slowing. Importantly, there was a significant negative correlation between response time and errors of commission (SATO) after soldiers completed the mentally fatiguing intervention, but not the control. As previously stated, the SATO in this marksmanship task typifies failures in response inhibition (speeded responses at the cost of accuracy) which have been attributed to central executive system overloading (Helton et al., 2010).

Mental fatigue appears to have differential effects on marksmanship performance. On the one hand mental fatigue had no measurable effects on marksmanship accuracy (i.e., hit proportion, DCSG, SGP) and correct shot response time. However, judgment accuracy (i.e., error of commissions) was significantly impaired which resulted in increased friendly-fire incidents. The discrepancy between these findings may be attributed to the fact that individual shot location relative to center aiming point was imperative to calculate the accuracy measures. Conversely, the judgment accuracy calculation was independent of whether the soldier hit the target or not (i.e., shots taken during inter-stimulus interval).

Interestingly, soldiers collectively did not rate the marksmanship tasks preceded by either of the interventions (mental fatigue vs. control), differently with respect to the global workload measure. Though speculative, there could be two reasons why there was not a difference in perceived workload. First, although the NASA-TLX is extensively used to measure workload, it may lack the sensitivity to adequately measure perceived workload of this task (Rubio et al., 2004). Alternatively, the lack of significant difference may suggest that the mental fatigue effect may be frontloaded at the start of the task and have a relatively short half-life. Given the design of the current study, we were not able to confidently extrapolate whether this assumption was correct. Future investigations should be designed to examine physical task performance as a function of time-on-task, instead of aggregate performance as previously done (e.g., Duncan et al., 2015; Head et al., 2016).

## Conclusion

The current study examined the effects of prior mental fatigue on subsequent marksmanship performance using a live-fire scenario with trained soldiers. The fatigue intervention was successful in eliciting mental fatigue. The results of the current investigation provide further support that mental fatigue modulates subsequent physical performance in a fine-motor task as similarly found in a full-body exercise task.

The findings of the current investigation may have practical implications for soldiers. For example, soldiers often have to perform vigilance type task that are mentally fatiguing such as stationary surveillance (e.g., sentry or security operations) or extended mobile patrols (e.g., monitoring environment in a mounted convoy or on dismounted patrol). Without warning, soldiers may need to engage multiple targets within an urban environment that contains a civilian population. Thus, soldiers need to not only be accurate and fast, but also judicious in their execution of sound judgment prior to pressing trigger. Anecdotally, soldiers have suggested that with modifications the marksmanship task used in this study could serve as an excellent training tool, both to decrease likelihood of errant fire upon friendly positions, as well as to improve soldier skill with respect to the speed of judicious target engagement. The task may hold particular relevance for contexts that feature commonality between adversarial and neutral/friendly visuo-perception characteristics, or that feature very short duration temporal constraints such as close-quarters (i.e., short distance-to-target) threats with concealed adversarial intent.

## Ethics statement

Ethics committee: The Human Research Engineering Directorate Institutional Review Board. Consent procedure: This study was carried out in accordance with the recommendations of name of guidelines, Human Research Engineering Directorate Institutional Review Board with written informed consent from all subjects. All subjects gave written informed consent in accordance with the Declaration of Helsinki. The protocol was approved by the Human Research Engineering Directorate Institutional Review Board. The informed consent explained the rational of the study and also risk involved by participating. Subjects were instructed that they could withdraw from the study at anytime for any reason without a negative consequence.

## Author contributions

JH: Substantially contributed to the conception, design, acquisition and analysis of data, and interpretation of the work, was involved in drafting the work, approved the final version to be published and agree to be accountable for all aspects of the work. MT: Highly contributed to the conception, design, acquisition, and analysis of data, and interpretation of the work, was involved in drafting the work, approved the final version to be published and agree to be accountable for all aspects of the work. AT: Highly contributed to revise the work critically for important intellectual content, approved the final version to be published, and agrees to be accountable for all aspects of the work. ML: Highly contributed to the conception, design, acquisition, and analysis of data, and interpretation of the work, was involved in drafting the work, approved the final version to be published and agree to be accountable for all aspects of the work. FM: Highly contributed to the conception, design, and interpretation of the work, was involved in drafting the work, approved the final version to be published and agrees to be accountable for all aspects of the work. KW, SO, and WH: Highly contributed to the conception, design, and interpretation of the work.

### Conflict of interest statement

The authors declare that the research was conducted in the absence of any commercial or financial relationships that could be construed as a potential conflict of interest.
